# Co-delivery of Cyclopamine and Doxorubicin Mediated by Bovine Serum Albumin Nanoparticles Reverses Doxorubicin Resistance in Breast Cancer by Down-regulating P-glycoprotein Expression: Erratum

**DOI:** 10.7150/jca.113959

**Published:** 2025-04-12

**Authors:** Yong-lin Lu, Ya-bin Ma, Chan Feng, Dong-lei Zhu, Jie Liu, Lv Chen, Shu-jing Liang, Chun-yan Dong

**Affiliations:** 1 Breast Cancer Center, Shanghai East Hospital, Tongji University, Shanghai 200120, PR China; 2 Department of Pharmacy, Shanghai East Hospital, Tongji University, Shanghai 200120, PR China

The original version of this article contained errors in Figure 7B. Specifically, three HE-stained images were incorrectly used; two images (heart and spleen) are from the DOX group, while one image (heart) is from the CYC+DOX group. These errors have now been corrected. All other images in the figure remain unchanged, and the interpretation of the results is unaffected. The authors apologize for any inconvenience this may have caused. The correct images appear below.

## Figures and Tables

**Figure 7:**
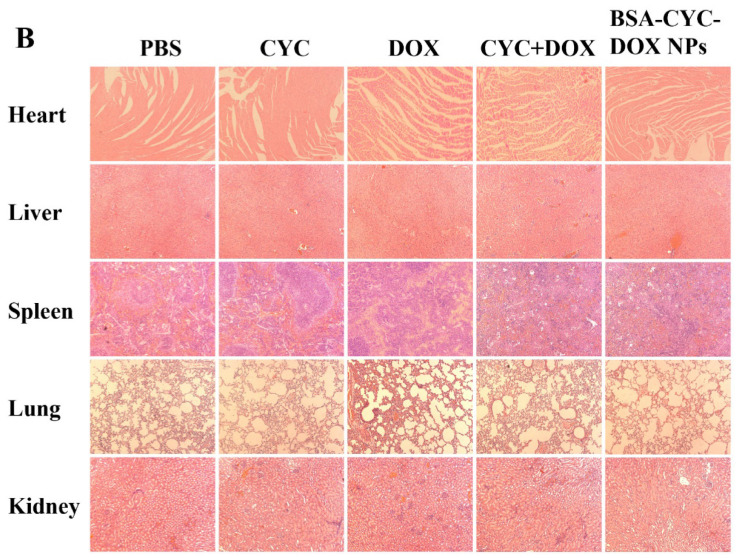
Histopathological examination of tumors and organs isolated from nude mice after different treatments on day 14. (b). H&E staining of main organs.

